# RNA-Seq Analysis Reveals the Role of Omp16 in *Brucella*-Infected RAW264.7 Cells

**DOI:** 10.3389/fvets.2021.646839

**Published:** 2021-03-04

**Authors:** Dong Zhou, Feijie Zhi, Jiaoyang Fang, Weifang Zheng, Junmei Li, Guangdong Zhang, Lei Chen, Yaping Jin, Aihua Wang

**Affiliations:** ^1^College of Veterinary Medicine, Northwest A&F University, Yangling, China; ^2^Key Laboratory of Animal Biotechnology of the Ministry of Agriculture, Northwest A&F University, Yangling, China

**Keywords:** *B. suis* S2, Omp16, RAW264.7, RNA-seq, interactions

## Abstract

Brucellosis is an endemic zoonotic infectious disease in the majority of developing countries, which causes huge economic losses. As immunogenic and protective antigens at the surface of *Brucella* spp., outer membrane proteins (Omps) are particularly attractive for developing vaccine and could have more relevant role in host–pathogen interactions. Omp16, a homolog to peptidoglycan-associated lipoproteins (Pals), is essential for *Brucella* survival *in vitro*. At present, the functions of Omp16 have been poorly studied. Here, the gene expression profile of RAW264.7 cells infected with *Brucella suis* vaccine strain 2 (*B. suis* S2) and ΔOmp16 was analyzed by RNA-seq to investigate the cellular response immediately after *Brucella* entry. The RNA-sequence analysis revealed that a total of 303 genes were significantly regulated by *B. suis* S2 24 h post-infection. Of these, 273 differentially expressed genes (DEGs) were upregulated, and 30 DEGs were downregulated. These DEGs were mainly involved in innate immune signaling pathways, including pattern recognition receptors (PRRs), proinflammatory cytokines, and chemokines by Kyoto Encyclopedia of Genes and Genomes (KEGG) analysis. In ΔOmp16-infected cells, the expression of 52 total cells genes was significantly upregulated and that of 9 total cells genes were downregulated compared to *B. suis* S2-infected RAW264.7 cells. The KEGG pathway analysis showed that several upregulated genes were proinflammatory cytokines and chemokines, such as interleukin (IL)-6, IL-11, IL-12β, C–C motif chemokine (CCL2), and CCL22. All together, we clearly demonstrate that ΔOmp16 can alter macrophage immune-related pathways to increase proinflammatory cytokines and chemokines, which provide insights into illuminating the *Brucella* pathogenic strategies.

## Introduction

As zoonotic pathogens, *Brucella* spp. cause a serious infection known as brucellosis that results in animal reproductive diseases and human chronic debilitating diseases (1, 2). A diverse array of land and aquatic mammals, including swine, cattle, goats, sheep, dogs, and dolphins, are known to serve as hosts for *Brucella* (1). It infects millions of livestock and more than half a million people annually, causing economic loss and a public health burden (2). Although brucellosis causes abortion and sterility in their hosts, the human disease is principally characterized by recurrent fever and osteoarticular complications during the chronic stage of the infection (3). In animals, live attenuated vaccines, including *Brucella abortus* S19 and *Brucella melitensis* Rev. 1, still have some disadvantages, such as serodiagnostic interference and residual pathogenicity (4–6). In China, live attenuated *Brucella suis* bv. 1 str. S2 vaccine (*B. suis* S2) is an essential and critical component in the control of brucellosis and also exhibits potential virulence reversion (7). Based on the lack of licensed human vaccines to protect against *Brucella*, safer and better vaccines need to be developed (4, 8).

*Brucella* outer membrane proteins (Omps) are important components of the cell wall (9). According to molecular weight of Omps, the *Brucella* cell Omps contains three major proteins ranging from 25 to 27, 31 to 34, and 36 to 38 kDa (10). At present, some experiments have shown that Omp10, Omp19, Omp25, and Omp31 are involved in *Brucella* virulence (11–13). The *Brucella* Omp19, Omp25, and Omp31 mutant were attenuated in cellular models and in mice, indicating that Omp19 and Omp25 were important for bacterial survival *in vitro* and *in vivo* (11, 14–17). Furthermore, Omp25 and Omp31 disrupt the immune response by regulating the secretion of tumor necrosis factor alpha (TNF-α) expression and apoptosis in porcine and murine macrophages infected models (18, 19). As the homolog of peptidoglycan-associated lipoproteins (Pals), Omp16 plays a vital role in the maintenance of membrane integrity and the import of certain organic molecules (20–22). Some experiments have shown that Omp16 was involved in *Brucella*-mediated immune response and can also be used as a protective antigen (23–26). However, attempt to directly delete Omp16 was unsuccessful, which also indicated that Omp16 is a vital gene for *Brucella* and plays an important role in the maintenance of membrane integrity and *Brucella* survival. In our previous study using an indirect method to tightly control Omp16 expression, *Brucella* cells lacking Omp16 presented defects in growth, outer membrane integrity, and intracellular survival (20). However, the role of Omp16 in *Brucella*–host interaction has not been well-studied.

In the present study, we identified 303 differentially expressed genes (DEGs) using RNA-seq in *B. suis* S2-infected RAW 264.7 cells compared to uninfected cells. In DEGs, most upregulated genes were involved in the immune system and cytokines, while downregulated genes were related to metabolism and cell cycle. On the basis of ATc-induced conditional complementation strain of the *B*. *suis* S2 Omp16, 61 DEGs were observed using RNA-seq in ΔOmp16-infected RAW 264.7 cells compared to *B. suis* S2-infected cells. The 52 upregulated genes were involving in pattern recognition receptors (PRRs) signaling pathway, including nucleotide oligomerization domain (NOD)-like receptor signaling pathway, chemokines, and cytokines, while 9 downregulated genes were related to metabolism. Real-time quantitative reverse transcription PCR (qRT-PCR) analysis further verified DEGs. The results presented here are expected to reveal the Omp16 roles during the *Brucella* infection process of RAW 264.7 cells and generate a new insight to explore the pathogenic mechanism of *Brucella*.

## Materials and Methods

### Bacteria Strains and Culture

In the present study, wild-type *B. suis* S2 (CVCC reference number, CVCC70502) bacteria strains were used. *B. suis* S2 ΔOmp16 have been constructed as described previously (20). Wild-type *B. suis* S2 and its derivatives were grown on tryptic soy agar (TSA; Sigma) for 72 h at 37°C or in tryptic soy broth (TSB) with shaking overnight to late-log growth phase. When indicated, bacteria cultures were treated with 50 μg/ml gentamicin and 50 μg/ml ampicillin. Then, wild-type *B. suis* S2 and its derivatives were collected by centrifugation, and the number of bacteria was confirmed using a 10-gradient dilution.

### Mammalian Cell Culture and Infection

RAW264.7 macrophage cells were cultured to monolayer in 6- or 24-well plates in Dulbecco's modified Eagle's medium (DMEM; Gibco; 1 g/L glucose) supplemented with 10% fetal bovine serum (FBS, Gibco) at 37°C with 5% CO_2_. For infection, RAW264.7 cells were seeded at 1 × 10^6^ cells/well (6-well plate) or 2 × 10^5^ cells/well (24-well plates) in complete medium 12 h before infection, then infected with wild-type *B. suis* S2 and its derivatives at the multiplicity of infection (MOI) of 200:1 for 4 h. Following 4 h of incubation at 37°C in 5% CO_2_, RAW264.7 cells were washed three times with 37°C phosphate-buffered saline (PBS) to remove extracellular *Brucella* and incubated for 1 h with medium supplemented with 50 μg/ml kanamycin to kill the remaining extracellular bacteria. Afterward, RAW264.7 cells were cultured in medium supplemented with 25 μg/ml kanamycin to avert continuous infection. This time was considered 0 h. RAW264.7 cells were collected for experiments at specific times.

### Collection of RAW264.7 Cells Samples for Transcription Analysis

*B. suis* S2 and ΔOmp16 were collected at late-log growth phase by centrifugation at 6,000 rpm for 5 min. The collected bacteria were washed three times with PBS, then suspended in PBS. The number of bacteria was confirmed using a 10-gradient dilution. RAW264.7 cells were infected with *B. suis* S2 or ΔOmp16 at MOI of 200:1; then, RAW264.7 cells were collected after 24 h with TRIzol RNA isolation reagent (Invitrogen, Inc., Carlsbad, CA, USA) for total RNA extraction.

### RNA-Seq Analysis

Total RNA was prepared as described. Using the Illumina Hiseq 2500 sequencer, RNA were sequenced separately. The reference genome data were downloaded from the National Center for Biotechnology Information (NCBI) database. Raw sequencing reads were cleaned by removing low-quality reads, reads containing poly-N sequences, and adaptor sequences. Then, clean reads were aligned to the reference genome using HISAT40. Using RESM software, the relative transcript abundance was calculated in fragments in reads per kilobase of exon sequence per million mapped sequence reads (FPKM). The *P* ≤ 0.05 and the absolute value of log2 ratio ≥1 were used to identify DEGs. The Gene Ontology (GO) database and KEGG database was used to analyze the pathway.

### Isolation of RNA From RAW264.7

According to the manufacturer's protocol, total RNA was extracted from *B. suis* S2- or ΔOmp16-infected RAW264.7 cells using TRIzol RNA isolation reagent (Invitrogen, Inc., Carlsbad, CA, USA). Total RNA quality and quantity were evaluated using the NanoDrop ND-1000 spectrophotometer (Thermo Scientific). RNA integrity was assessed by standard denaturing 1% agarose gel electrophoresis.

### Quantitative Real-Time PCR

To validate the data generated from the RNA-seq experiment, 13 pathway genes were selected to further analyze via quantitative real-time PCR (qRT-PCR). Total RNA were prepared as described. Briefly, RNA was reverse transcribed into complementary DNA (cDNA) using Maxima First-Strand cDNA synthesis kit (Thermo Fisher Scientific) according to the manufacturer's protocol. qRT-PCR was performed using SYBR Premix Ex Taq™ (Vazyme) and an ABI 7500 Sequencing Detection System. Using the 2^−Δ*ΔCt*^ method, qRT-PCR data were normalized, and glyceraldehyde 3-phosphate dehydrogenase gene (GAPDH) was used an as internal control. All the primers was designed according to mouse messenger RNAs (mRNAs) and are listed in Supplementary Table 1.

### Statistical Analysis

SPSS software was used for all statistical analyses (version 22; SPSS, Inc., Chicago, IL). All results were repeated at least three times and are presented as the means ± standard deviations (SDs). An unpaired, two-tailed Student's *t*-test or one-way analysis with group comparisons was used. A probability (*P*) value of ≤ 0.05 was considered significant.

## Results

### RNA Quality Analysis and Global Array Data

RNA integrity was determined via denatured agarose gel electrophoresis. Purity and concentration were measured using a spectrophotometer. Electrophoresis showed distinct three bands of 5S, 16S, and 23S ribosomal RNA (rRNA), indicating that the total RNA of RAW 264.7 cells were intact. Spectrophotometric RNA analysis revealed an OD_260_/OD_280_ ratio of >1.8, indicating superior quality of the RNA samples suitable for the RNA-seq analysis.

**Figure 1:**
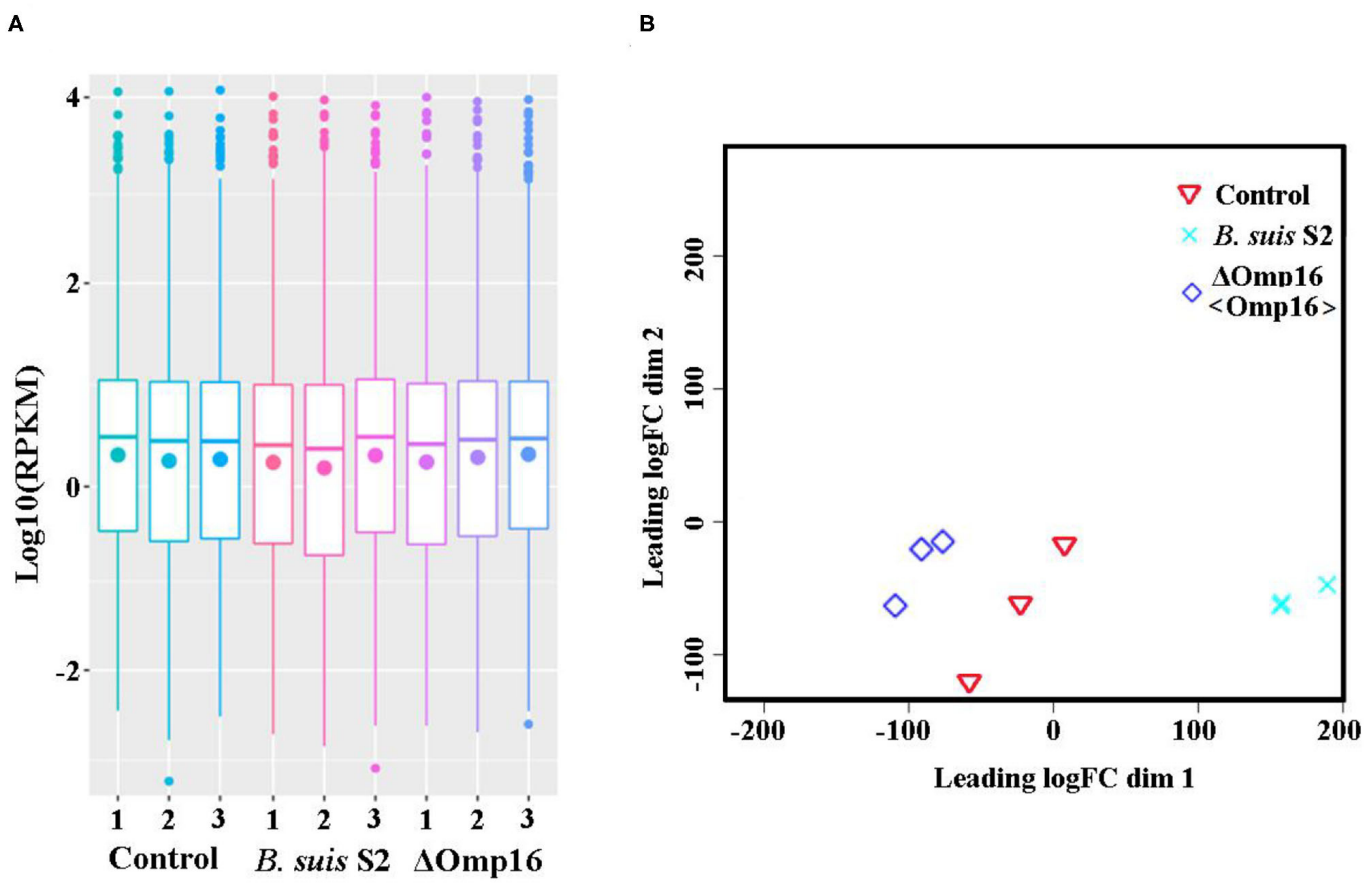
Assessment of gene data quality of all samples. **(A)** A box plot used to compared the intensity distribution of all bacterial samples. The distributions of log_10_ [reads per kilobase per million mapped reads (RPKM)] ratios among bacterial samples are nearly the same; **(B)** similarities visualized among bacterial samples using an multidimensional scaling (MDS) analysis. Red: uninfected RAW 264.7 cells. Green: *B. suis* S2-infected RAW 264.7 cells. Blue: ΔOmp16-infected RAW 264.7 cells.

Using RNA-seq, we conduct a comprehensive comparative transcriptomic analysis of the gene expression profiles of the uninfected, *B. suis* S2-infected-, and ΔOmp16-infected-RAW 264.7 cells. The major characteristics of these libraries for each group are presented in Table 1. The box plot was used to evaluate the intensity distribution of all samples. The distributions of log_10_ (reads per kilobase per million mapped reads, RPKM) among the uninfected, *B. suis* S2-infected-, and ΔOmp16-infected-RAW 264.7 cells were similar (Figure 1A). In addition, the multidimensional scaling (MDS) analysis was used to evaluate the biological repetition of all samples, indicating that three groups samples have a high reproducibility of the data (Figure 1B).

**Table 1:** Major characteristics of mRNA libraries and database generated by RNA-seq.

**Sample**	**Raw bases (G)**	**Raw reads**	**Clean reads**	**Total mapped (%)**
Control-1	6.72	44779952	42686754	92.68
Control-2	8.58	57180964	54654018	92.81
Control-3	7.62	50818606	48638136	92.31
*B. suis*. S2-1	6.51	43430156	41599652	93.16
*B. suis*. S2-2	9.27	61783014	58951842	92.92
*B. suis*. S2-3	7.72	51497980	48974982	92.26
ΔDnaA < DnaA>-1	5.83	38846290	37181292	92.33
ΔDnaA < DnaA>-2	6.99	46600322	44546310	92.83
ΔDnaA < DnaA>-3	6.49	43256424	41389296	92.29

### Determination of DEGs Between in Uninfected Cells and *B. suis* S2-Infected Cells

**Figure 2:**
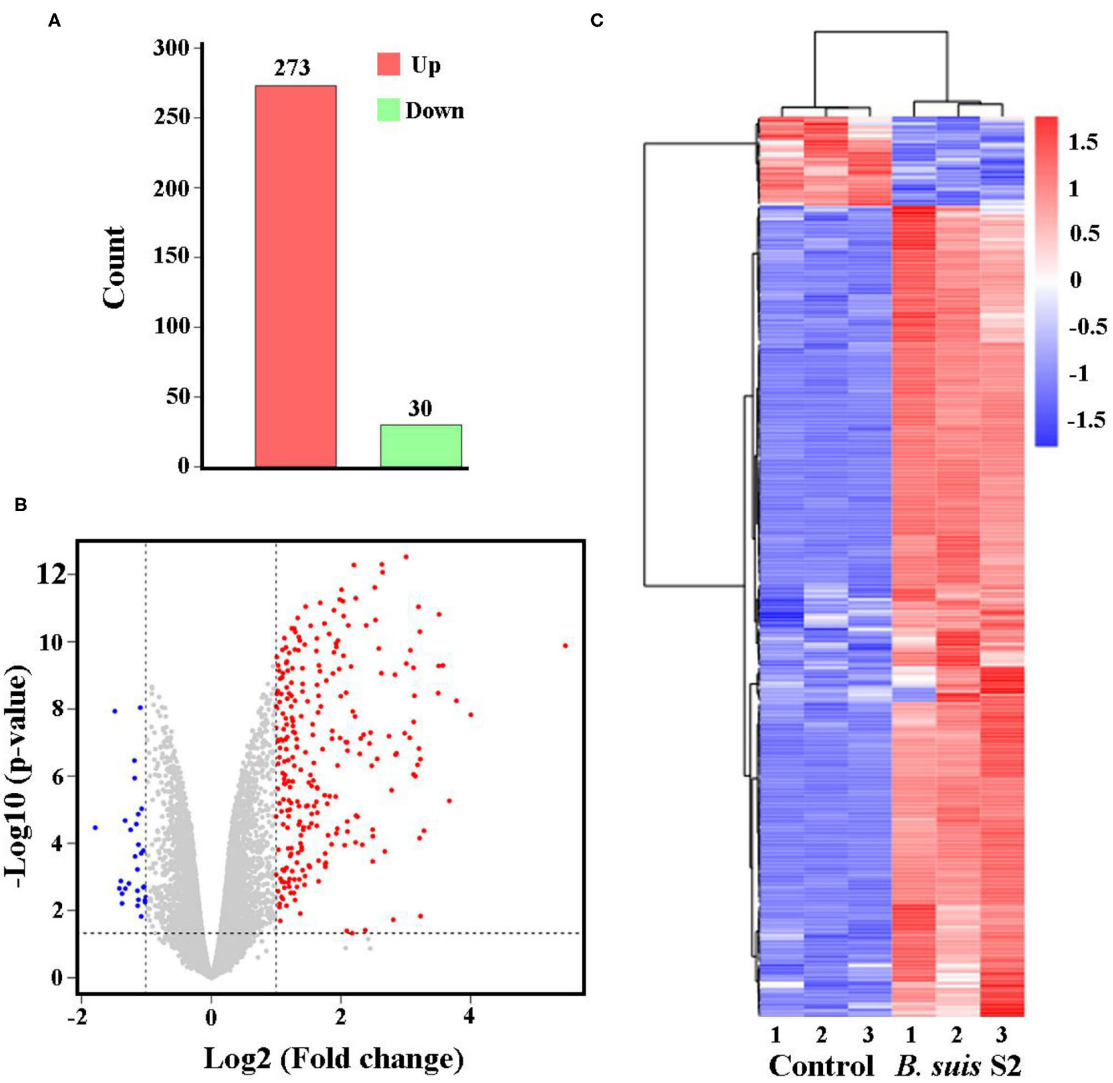
Expression of differentially expressed genes (DEGs) between the uninfected and *B. suis* S2-infected cells. **(A)** Histogram shows the number of DEGs and non-regulated genes between the between the uninfected and *B. suis* S2-infected cells. **(B)** Scatter plot of coexpressed genes between the between the uninfected and *B. suis* S2-infected cells. The red, blue, and gray colors denote upregulated, downregulated, and non-regulated genes, respectively, in the *B. suis* S2-infected cells compared with the uninfected cells based on the following criteria: absolute log2 (fold change) ≥1 and adjusted *P* ≤ 0.05. **(C)** The heatmap shows the expression levels of DEGs between the uninfected and *B. suis* S2-infected cells.

The gene expression profiles were compared between uninfected and *B. suis* S2-infected RAW 264.7 cells, and the whole gene expression levels were analyzed by Illumina HiSeq^TM^ 2500. Our comparative transcriptomic analysis revealed 303 DEGs [false discovery rate (FDR) <0.05, fold change ≥2]. Of the 303 DEGs, 273 genes were upregulated and 30 genes were downregulated in *B. suis* S2-infected RAW 264.7 cells compared to uninfected RAW 264.7 cells (Figure 2A and Supplementary Datasheet 1). Furthermore, 303 DEGs were shown via Volcano Plot between uninfected and *B. suis* S2-infected RAW 264.7 cells (Figure 2B). To analyze gene expression data, hierarchical clustering is widely used. On the basis of their expression levels, cluster analysis arranges samples into groups to elucidate possible relationships among samples. In our study, DEGs were analyzed by cluster analysis. A heatmap of these DEGs was presented (Figure 2C).

**Figure 3:**
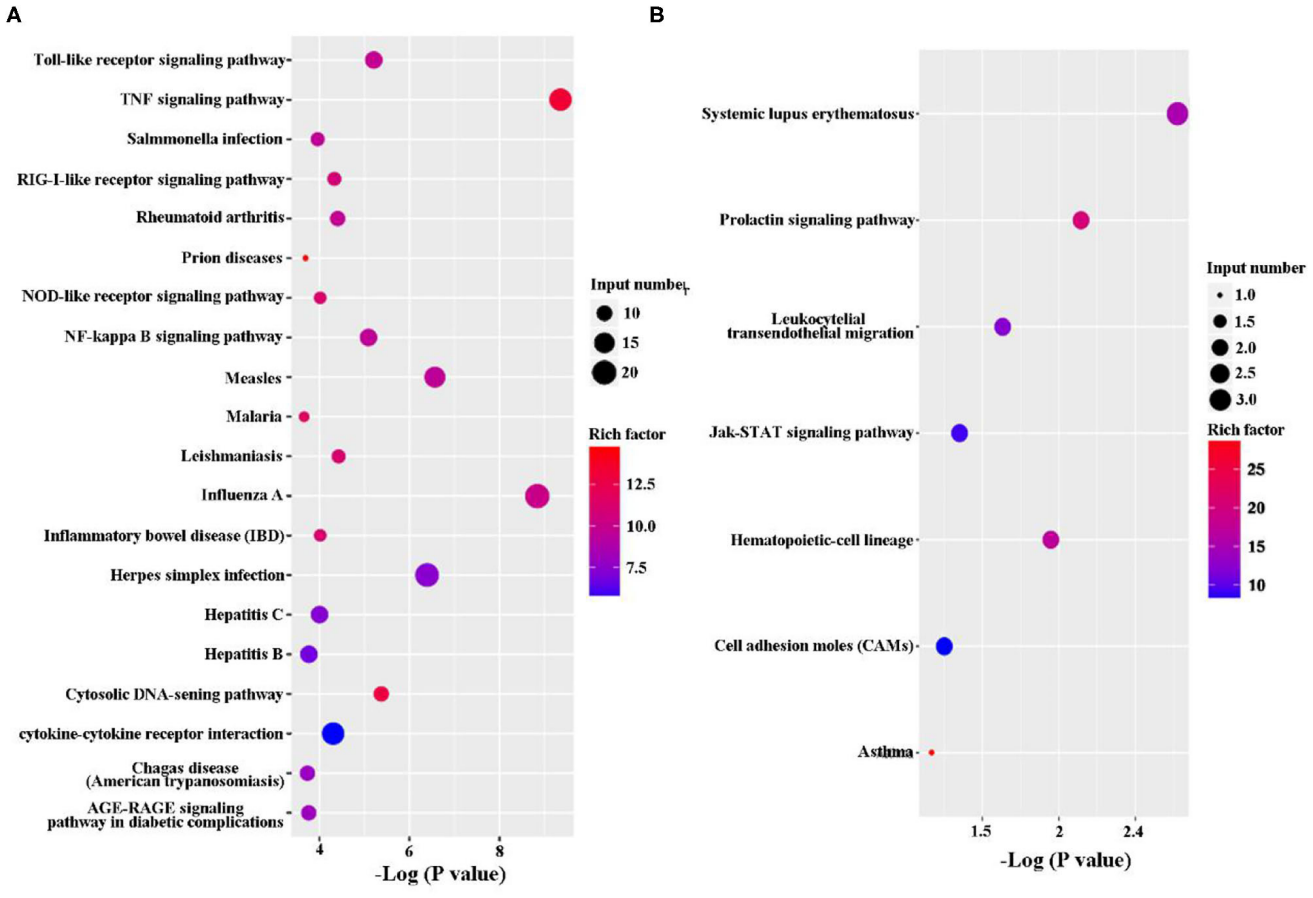
Kyoto Encyclopedia of Genes and Genomes (KEGG) classification of differentially expressed genes (DEGs) between the uninfected and *B. suis* S2-infected cells. The rich factor represents the ratio of **(A)** upregulated genes and **(B)** downregulated genes differentially expressed gene numbers annotated in this pathway term to all gene numbers annotated with this pathway term. A greater rich factor indicates a greater degree of pathway enrichment. The *Q*-value represents the corrected *P*-value and ranges from 0 to 1, and a lower value indicates greater pathway enrichment.

The KEGG pathway enrichment analysis was performed to analyze DEGs. Based on KEGG pathway enrichment analysis, a majority of the most upregulated genes were involved in immune response, including PRRs (Toll-like receptor signaling pathway and NOD-like receptor signaling pathway), cytokines (IL-1, IL-6, IL-23, and Cfs3), and chemokines (Ccl2, Ccl3, Ccl4, Ccl5, and Ccl10; Figure 3A and Supplementary Datasheet 2). In addition, apoptosis-related genes, such as TNF, Traf1, Nfkbia, Bcl2, and Gadd45b, were upregulated (Figure 3A and Supplementary Datasheet 2). However, the major downregulated genes were involved in metabolism and proliferation, including Rapgef3, St6gal1, Cd109, Cish, Gm17041, and Cd24a (Figure 3B and Supplementary Datasheet 2).

### Determination of DEGs Between in *B. suis* S2-Infected Cells and ΔOmp16-Infected Cells

**Figure 4:**
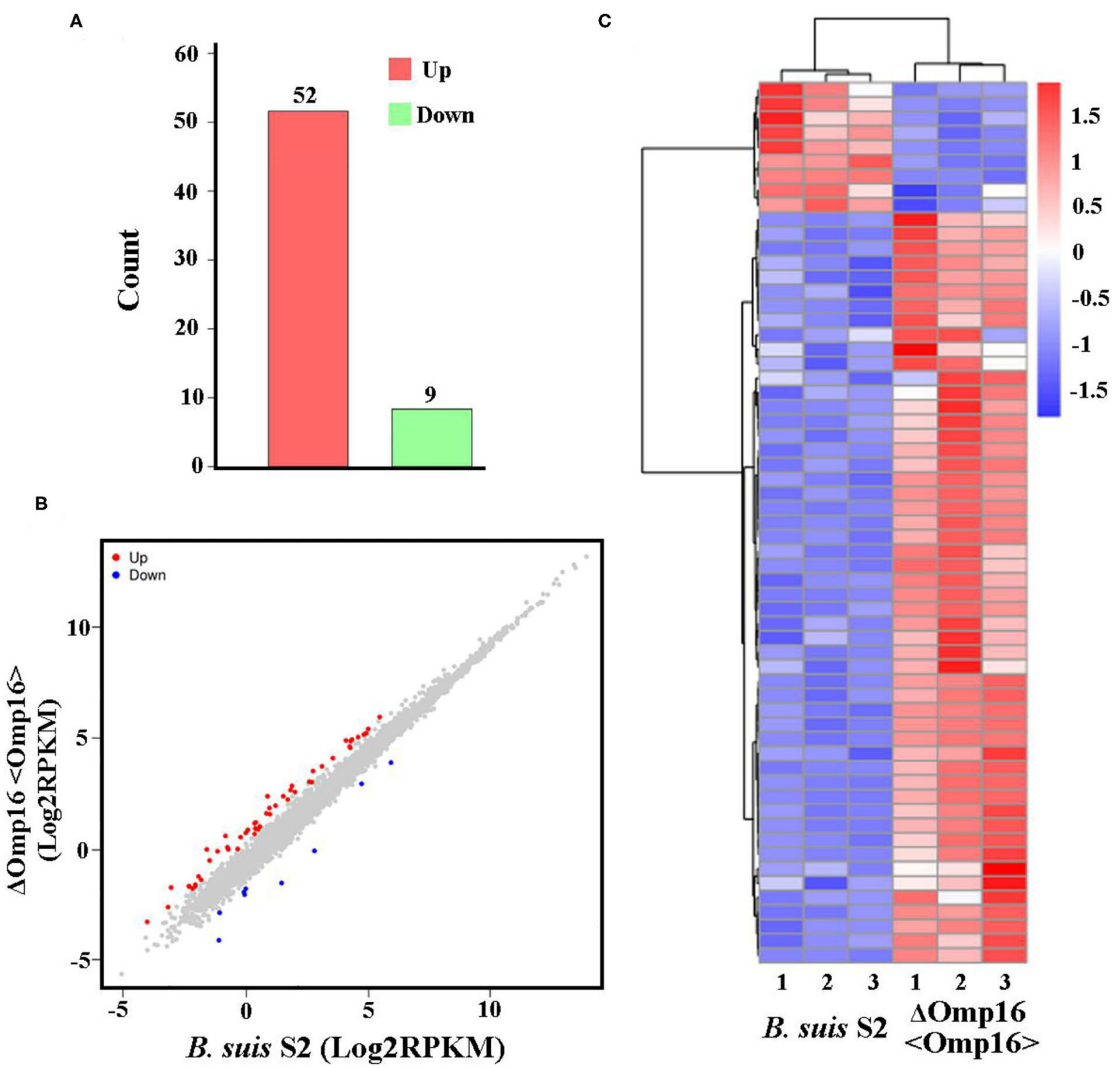
Expression of differentially expressed genes (DEGs) between *B. suis* S2-infected and ΔOmp16-infected cells. **(A)** Histogram shows the number of DEGs and non-regulated genes between *B. suis* S2-infected and ΔOmp16-infected cells. **(B)** Scatter plot of coexpressed genes between the between *B. suis* S2-infected and ΔOmp16-infected cells. The red, blue, and gray colors denote upregulated, downregulated, and non-regulated genes, respectively, in the *B. suis* S2-infected cells compared with the uninfected cells based on the following criteria: absolute log2 (fold change) ≥1 and adjusted *P* ≤ 0.05. **(C)** The heatmap shows the expression levels of DEGs between the between *B. suis* S2-infected and ΔOmp16-infected cells.

Multiple attempts to delete Omp16 were unsuccessful in *Brucella ovis* PA (13). We also made several attempts to delete Omp16 in *B. suis* S2 strain but were unsuccessful, which indicated that Omp16 could be a vital gene. Therefore, we obtained ΔOmp16 strain via conditional complementation using tetracycline-induced gene expression system (20). On the basis of ΔOmp16 strain, the gene expression profiles were compared between *B. suis* S2-infected RAW 264.7 cells and ΔOmp16-infected RAW 264.7 cells, and the whole gene expression levels were analyzed by Illumina HiSeq^TM^ 2500. We revealed 61 DEGs (FDR <0.05, fold change ≥2) via RNA-seq. Compared to *B. suis* S2-infected RAW 264.7 cells, 52 genes were upregulated and 9 genes were downregulated among the 61 DEGs in ΔOmp16-infected RAW 264.7 cells (Figure 4A and Supplementary Datasheet 3). Moreover, 61 DEGs were shown via Volcano Plot between *B. suis* S2- and ΔOmp16-infected RAW 264.7 cells (Figure 4B). In addition, a heatmap of the DEGs was drawn to directly observe the DEGs expression (Figure 4C).

**Figure 5:**
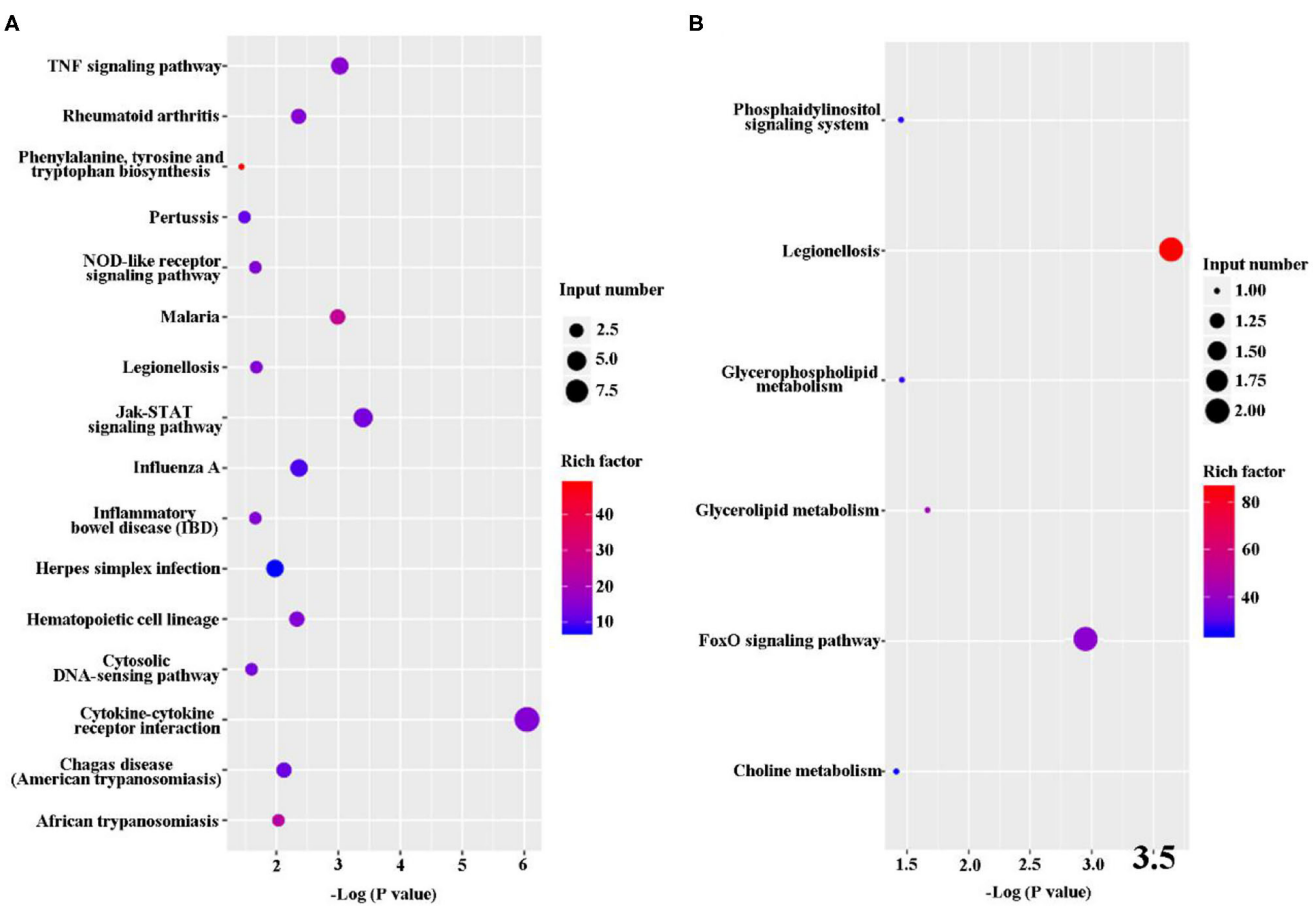
Kyoto Encyclopedia of Genes and Genomes (KEGG) classification of differentially expressed genes (DEGs) between *B. suis* S2-infected and ΔOmp16-infected cells. The rich factor represents the ratio of **(A)** upregulated genes and **(B)** downregulated genes differentially expressed gene numbers annotated in this pathway term to all gene numbers annotated with this pathway term. A greater rich factor indicates a greater degree of pathway enrichment. The *Q*-value represents the corrected *P*-value and ranges from 0 to 1, and a lower value indicates greater pathway enrichment.

The DEGs were analyze via KEGG pathway enrichment analysis. On the one hand, a majority of the upregulated genes, including Tnfrsf8, Ccl2, IL-12β, IL-11, Ccl22, Csf3, Lif, Tnfrsf1b, and IL-6, were related to immune response, such as TNF signaling pathway, NOD-like receptor signaling pathway, Jak-STAT signaling pathway, cytosolic DNA-sensing pathway, and cytokine–cytokine receptor interaction (Figure 5A and Supplementary Datasheet 4). On the other hand, the downregulated genes, such Bnip3 Gm45507 and Dgkg, were involved in phosphatidylinositol signaling system, legionellosis, glycerophospholipid metabolism, glycerolipid metabolism, FoxD signaling pathway, and choline metabolism (Figure 5B and Supplementary Datasheet 4).

### qRT-PCR Verification of the RNA-Seq Results

**Figure 6:**
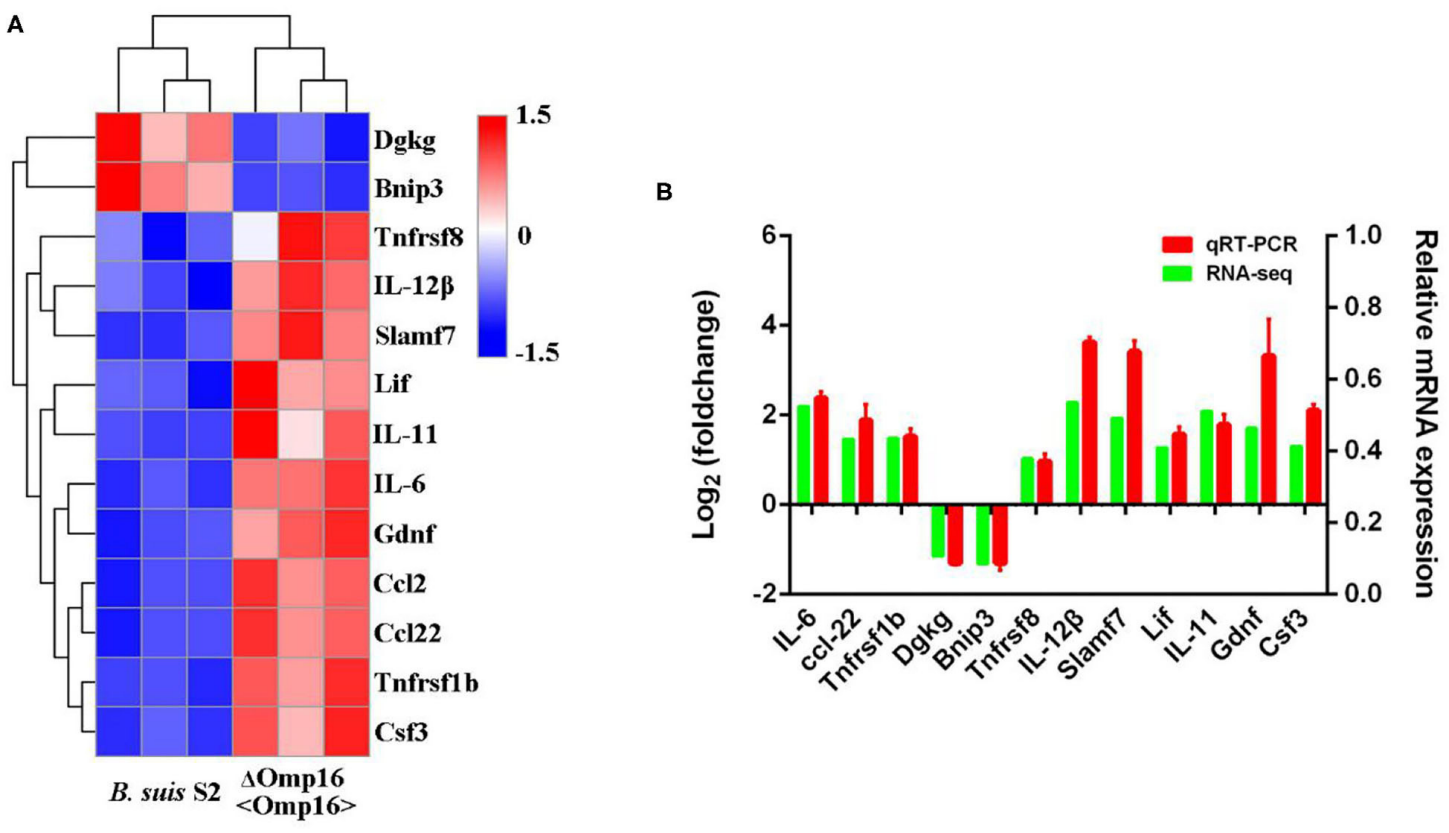
Differentially expressed genes (DEGs) were evaluated by quantitative reverse transcription PCR (qRT-PCR) assays between *B. suis* S2-infected and ΔOmp16-infected cells. **(A)** The heatmap shows the expression levels of 13 DEGs between the between *B. suis* S2-infected and ΔOmp16-infected cells. **(B)** The 10 DEGs expression levels were further detected by qRT-PCR. The results at each time point are expressed as the means ± standard deviations from at least three independent experiments.

In order to validate the RNA-seq data and ensure technical reproducibility, we selected and evaluated expression of 11 upregulated genes (Gdnf, Ccl2, IL-12β, IL-11, Ccl22, Csf3, Lif, Tnfrsf1b, Tnfrsf8, Slamf7, and IL-6) and 2 downregulated genes (Dgkg and Bnip3) from ΔOmp16-infected RAW 264.7 cells by qRT-PCR. The expressions of these genes obtained using qRT-PCR were in good agreement with the RNA-seq results (Figures 6A,B).

## Discussion

It has long been recognized that *Brucella* interaction with macrophages is the key aspect of its pathogenesis (2). *Brucella* can elude initial innate immune recognition through modifications of virulence factors such as lipopolysaccharide (LPS) and flagellin, resulting in a mild proinflammatory response that leads to bacterial persistence (2). However, the effects of Omps on host–pathogen interactions have not been fully understood.

In this study, we conducted a comparative transcriptomic analysis among uninfected, *B. suis* S2-infected, or ΔOmp16-infected RAW 264.7 cells to reveal the role of Omp16 during *Brucella*-infected RAW 264.7 cells. After being challenged with *B. suis* S2, we found that a majority of the most upregulated genes were involved in immune response, including PRRs (Toll-like receptor signaling pathway and NOD-like receptor signaling pathway), cytokines (IL-1, IL-6, IL-23, and Cfs3), and chemokines (Ccl2, Ccl3, Ccl4, Ccl5, and Ccl10), but the major downregulated genes were involved in metabolic and proliferation, including Rapgef3, St6gal1, Cd109, Cish, Gm17041, and Cd24. On this basis, we used ΔOmp16 that were previously constructed via conditional complementation by ATc. Compared to *B. suis* S2-infected RAW 264.7 cells, 61 DGEs were found in ΔOmp16-infected RAW 264.7 cells. Surprisingly, some immune-function-related genes were upregulated and were involved in multiple signaling pathways, such as TNF signaling pathway, NOD-like receptor signaling pathway, Jak-STAT signaling pathway, cytosolic DNA-sensing pathway, and cytokine–cytokine receptor interaction. In conclusion, these data provided evidence that Omp16 plays an important role in *Brucella*-induced immune response during infection.

During infection, the host is able to quickly detect invading pathogens to induce immune response to remove invasive pathogens, including initial inflammatory response (2, 3, 27). As a facultative intracellular pathogen, *Brucella* uses multiple strategies to escape immune defense mechanism of the host for survival, such as evading detection by pathogen-associated molecular patterns (PAMPs) (2, 28), inhibiting apoptosis, downregulating antigen presentation, and so on (29). In RNA-seq data, some upregulated genes were involved in PRR signaling pathway, including Toll-like receptor signaling pathway (TLRs) and NOD-like receptor signaling pathway (NLRs), indicating that *Brucella* can activate the host's innate immune response. However, the activation was very weak. *Brucella*, a chronic pathogen, has developed special mechanisms to evade TLR and NLR detection to maintain persistent infection. *Brucella* limits the cell's TLR4 detection of LPS with a longer fatty-acid chain, resulting in a mild proinflammatory response (30). *Brucella*-regulated flagellin synthesis to limit TLR5 detection is the stealthy strategy of *Brucella* toward the innate immune system (31). In addition, *Brucella* is able to degrade MyD88 adaptor-like (MAL) by secreting effector proteins that contain a Toll-interleukin-1 receptor (TIR) domain, such as BtpA and BtpB (1, 32, 33).

The intracellular nature of *Brucella* spp. makes it difficult to eliminate these bacteria by antimicrobial response drugs (29, 34). Thus, several cytokines and chemokines are key players against brucellosis, inducing innate and adaptive immune responses (3). The adaptive immune response to *Brucella* spp. is characterized by elevated levels of proinflammatory cytokines linked to Th1 responses, such as IL-1β and IL-6 (2, 3). In RNA-seq, compared to uninfected group, the Th1-responses-related cytokines, including IL-1 and IL-6, were increased in *B. suis* S2-infected cells, indicating that *Brucella* was able to activate RAW 264.7 macrophage cells to produce Th1 response-related cytokines. In addition, NF-kB, a central transcription factor, was responsible for controlling about 150 target genes expression, including multiple cytokines, chemokines, and receptors required for immune recognition (35). Thus, the NF-kB signaling pathway plays an important role in resistance to *Brucella* infection. In RNA-seq, KEGG pathway enrichment analysis shows that upregulated gene is enriched in the NF-kB signaling pathway, indicating that NF-kB signaling pathway is involved in eliminating intracellular *Brucella*.

*Brucella* spp. Omps have been broadly characterized as immunogenic and protective antigens (36, 37). Omp16, a homolog to Pals, is vital for *Brucella* survival *in vivo* or *in vitro* (13, 20). Compared to *B. suis* S2-infected RAW 264.7 cells, some inflammatory cytokines were upregulated, including IL-6, IL-11, and IL-12β, indicating that Omp16 could inhibit some inflammatory cytokines to promote *Brucella* intracellular survival. The mRNA expression of IL-6 was enhanced in ΔOmp16-infected RAW 264.7 cells compared to *B. suis* S2-infected RAW 264.7 cells (20). These results are consistent with the RNA-seq results. In previous studies, IL-6 contributes to increasing susceptibility during infection (38, 39). *Brucella* have some Omps that inhibit several cytokine secretions to contribute to intracellular survival. In porcine and murine macrophages, *Brucella* Omp25 inhibited TNF-α expression to promote intracellular survival via regulating different microRNA (18).

Interestingly, metabolic and proliferation-related genes are downregulated in RNA-seq, indicating that the activity of RAW 264.7 cells is decreased during *Brucella* infection. In the past, studies were mainly focused on the pathogen intracellular survival, inflammation response, immune response, and apoptosis. Thus, exploring the role of metabolic and proliferation-related genes is required.

## Conclusion

The RNA-sequence analysis revealed that 303 genes were significantly regulated by *B. suis* S2, and these DEGs were mainly involved in innate immune signaling pathways, including PRRs and proinflammatory cytokines and chemokines. In ΔOmp1-infected RAW 264.7 cells, the expressions of 52 total cell genes were significantly upregulated and that of 9 total cells genes were downregulated. The KEGG pathway analysis showed that several upregulated genes were proinflammatory cytokines and chemokines. All together, we clearly demonstrate that ΔOmp16 can alter macrophage immune-related pathways to increase proinflammatory cytokines and chemokines. Further deep understanding of the regulation mechanisms of Omp16 in *Brucella*-infected macrophage may help to provide insights into illuminating the *Brucella* pathogenic strategies.

## Data Availability Statement

The datasets presented in this study can be found in online repositories. The names of the repository/repositories and accession number(s) can be found below: Dryad doi: 10.5061/dryad.tqjq2bvxv.

## Author Contributions

AW contributed to the conception and design of the work. FZ, JF, JL, GZ, and LC executed the experiments. DZ and FZ contributed to the analysis and interpretation of the data and drafted the manuscript. YJ and AW contributed to the final approval of the version for publication. All authors have read and agreed to the published version of the manuscript.

## Conflict of Interest

The authors declare that the research was conducted in the absence of any commercial or financial relationships that could be construed as a potential conflict of interest.

## Supplementary Material

Supplementary Table 1 A list of all primers used in qRT-PCR.

Supplementary Datasheet 1 The DEGs between the uninfected and *B. suis* S2-infected cells.

Supplementary Datasheet 2 The DEGs KEGG classification between the uninfected and *B. suis* S2-infected cells.

Supplementary Datasheet 3 The DEGs between *B. suis* S2-infected and ΔOmp16-infected cells.

Supplementary Datasheet 4 The DEGs KEGG classification between *B. suis* S2-infected and ΔOmp16-infected cells.
