# Supplementary material for: RNA-Seq Analysis Reveals the Role of Omp16 in Brucella-Infected RAW264.7 Cells
Source: Front Vet Sci. 2021 Mar 4;8:646839. doi: 10.3389/fvets.2021.646839 (PMC7970042; doi:10.3389/fvets.2021.646839)
Supplement: Supplementary Table 1 — A list of all primers used in qRT-PCR. [file Table_1.docx]

| Gene | Forward primer(5'-3') | Reverse primer(5'-3') |
| --- | --- | --- |
| Dgkg | TGAAGGTGGAAGCCTCAAGC | TCTTGTCTGGGCTTCTGGC |
| Bnip3 | GCTCCAAGAGTTCTCACTGTGAC | GTTTTTCTCGCCAAAGCTGTGGC |
| Tnfrsf8 | ATGAAGACGGGAAGTGCACAG | CGGAACACGGAGCCTTCTC |
| IL-12β | CAGAAGCTAACCATCTCCTGGTTTG | TCCGGAGTAATTTGGTGCTTCACAC |
| Slamf7 | AATGGCACCTGCGTAATC | GTGTCATAGTCTGCGTTCT |
| Lif | CTTCTCCCTCTGGTCTCCAA | GGGTCAGGATGTTTCAGCAC |
| Gdnf | CGCTGACCAGTGACTCCAATATGC | TGCCGCTTGTTTATCTGGTGACC |
| Tnfrsf1b | CAGGTTGTCTTGACACCCTAC | GCACAGCACATCTGAGCCT |
| Csf3 | TTGGCAACATCCAGCTGAAG | GCAGGCTCTATCGGGTATTTCC |
| Ccl22 | AAGCCTGGCGTTGTTTTGAT | CCTGGGATCGGCACAGATA |
| IL-6 | GATGCTACCAAACTGGATATAATC | GGTCCTTAGCCACTCCTTCTGTG |
| IL-11 | GCTGTTCTCCTAACCCG | CTGGCTCCAGAGTCTT |
| Ccl2 | GAGGAAGGCCAGCCCAGCAC | TGGATGCTCCAGCCGGCAAC |

**Table S1 A list of all primers used in qRT-PCR**
